# CRISPR-induced RASGAP deficiencies in colorectal cancer organoids reveal that only loss of NF1 promotes resistance to EGFR inhibition

**DOI:** 10.18632/oncotarget.26677

**Published:** 2019-02-15

**Authors:** Jasmin B. Post, Nizar Hami, Alexander E.E. Mertens, Suraya Elfrink, Johannes L. Bos, Hugo J.G. Snippert

**Affiliations:** ^1^ Center for Molecular Medicine, Section Molecular Cancer Research, University Medical Center Utrecht, Utrecht, The Netherlands; ^2^ Oncode Netherlands, Institute Netherlands, Office Jaarbeurs Innovation Mile, Utrecht, The Netherlands

**Keywords:** NF1, RASGAP, anti-EGFR therapy resistance, cancer progression, colorectal cancer

## Abstract

Anti-EGFR therapy is used to treat metastatic colorectal cancer (CRC) patients, for which initial response rates of 10–20% have been achieved. Although the presence of HER2 amplifications and oncogenic mutations in KRAS, NRAS, and BRAF are associated with EGFR-targeted therapy resistance, for a large population of CRC patients the underlying mechanism of RAS-MEK-ERK hyperactivation is not clear. Loss-of-function mutations in RASGAPs are often speculated in literature to promote CRC growth as being negative regulators of RAS, but direct experimental evidence is lacking. We generated a CRISPR-mediated knock out panel of all RASGAPs in patient-derived CRC organoids and found that only loss of NF1, but no other RASGAPs e.g. RASA1, results in enhanced RAS-ERK signal amplification and improved tolerance towards limited EGF stimulation. Our data suggests that NF1-deficient CRCs are likely not responsive to anti-EGFR monotherapy and can potentially function as a biomarker for CRC progression.

## INTRODUCTION

Colorectal cancer (CRC) is one of the most common cancers and the third leading cause of worldwide cancer deaths (IARC). The use of monoclonal antibodies (moAbs) targeting the epidermal growth factor receptor (EGFR), such as cetuximab and panitumumab, together with chemotherapy has shown a clinical benefit for the treatment of patients with metastatic CRC (mCRC) [1–3]. The binding of these antibodies to the extracellular domain of the EGFR inhibits downstream activation of the RAS-MEK-ERK signaling pathway, thereby inhibiting cell proliferation and survival [4, 5]. Treatment with EGFR targeting moAbs resulted in initial response rates of 10–20% in mCRC patients [1], but it soon became clear that tumors with activating mutations in KRAS showed resistance to EGFR inhibition [2, 3, 6]. Moreover, the treatment of patients with mutant KRAS colorectal tumors with EGFR inhibitors seemed to aggravate disease progression [3]. Therefore these patients are now being excluded from EGFR targeted therapy [7, 8].

RAS proteins act as molecular switches that cycle between inactive GDP-bound and active GTP-bound states. Active GTP-bound RAS can stimulate a large variety of downstream signaling cascades, including the mitogen activated protein kinase (MAPK) and phosphatidylinositol-3-kinase (PI3K) pathways, to promote proliferation, migration and survival. The activation of RAS is tightly regulated by guanine nucleotide exchange factors (GEFs) and GTPases activating proteins (GAPs). GEFs accelerate the dissociation of GDP from RAS, whereas GAPs enhance the intrinsic rate of GTP hydrolysis of RAS [9]. Activating mutations in KRAS are identified in approximately 35-50% of mCRC patients, resulting in the constitutive downstream activation of MEK and ERK [9, 10]. It is thought that KRAS oncogenic mutations are early events in cancer progression, potentially even at the onset of tumorigenesis, as they are frequently found in both early and late stages of CRC [10–12]. In agreement with this, genomic studies have highlighted that the MAPK signaling pathway is often aberrantly activated in colorectal tumors [13–16]. However, in contrast to pancreatic cancers, where oncogenic KRAS mutations are found in 90% of the cases [17, 18], a large population of CRC patients carry tumors that are wild type for KRAS. Indeed, other oncogenic mutations in the MAPK signaling pathway, such as mutations in NRAS, BRAF, or HER2 amplifications, have been identified in CRC and are implicated in tumor progression [19–23]. Nevertheless, for at least 25% of mCRC patients the underlying cause of aberrant MAPK pathway activation remains unknown [19–22].

In this regard, RASGAPs that act as negative regulators of RAS signaling are frequently implicated in tumorigenesis. In the human genome, ten functional RASGAP genes have been identified. Genetic analysis of tumor samples only identified a significant number of inactivating mutations in the RASGAPs neurofibromin (NF1) and RASA1 (p120GAP), suggesting that these two RASGAPs can function as tumor suppressors. Moreover, ongoing sequencing efforts of larger patient cohorts may increase the detection of low abundant loss-of-function mutations in several other RASGAPs [24, 25].

Loss-of-function mutations in *NF1* are frequently associated with a large variety of cancers, such as melanoma [26–29], leukemia [30–32], glioblastoma [33], and lung cancer [25]. Moreover, multiple studies have linked NF1 activity to RAS and ERK activity [28, 29, 33–36], including its role in therapy resistance upon targeted inhibition of the MAPK pathway in melanoma [28, 29, 36, 37] and lung cancer [38]. Inactivating mutations and deletions in the *RASA1* gene have also been detected in a number of cancers, such as lung squamous carcinoma [39], stomach, esophagus [40], leukemia [41], and head and neck [25] cancer, but its role as a tumor suppressor is less well defined.

In line with their molecular function, a suggestive tumor suppressive role for RASGAPs in CRC has been proposed based on association studies [42–46], as well as knock-down experiments in cell lines [47, 48]. However, the debate whether indeed all RASGAPs can mediate CRC progression beyond EGF dependence remains ongoing, in particular since the lack of direct loss-of-function data regarding RASGAPs in CRC models.

Here, using CRISPR-mediated knock out lines in patient-derived CRC organoids that are otherwise wild type for the RAS pathway, we investigate the role of RASGAPs in CRC progression and in relation to EGFR signaling. Surprisingly, in contrast to widely accepted assumptions, but in line with overall mutation frequencies, we show that only the loss of NF1, but no other RASGAPs, can act as an amplifier of MAPK signaling. As such, NF1-deficiency contributes to CRC progression by minimizing its dependence on EGF-ligand stimulated MAPK signaling.

## RESULTS

### Low abundant mutation frequencies for RASGAPs in CRC

Strong activating mutations of RAS pathway effectors tend to occur in a mutually exclusive manner, most pronounced for oncogenic mutations in either *RAS* or *BRAF*.

**Figure 1:**
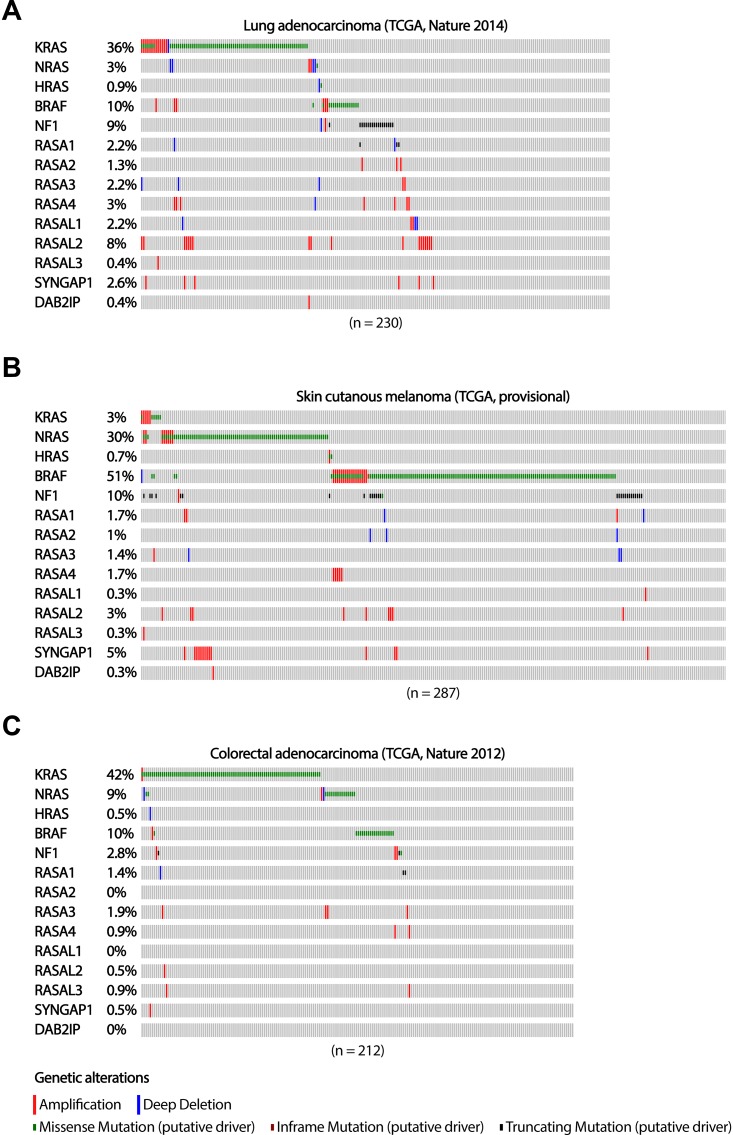
The occurrence of RASGAP and oncogenic mutations in the MAPK signaling pathway in lung adenocarcinoma, melanoma and colorectal adenocarcinoma The distribution of driver mutations and copy number alterations in *KRAS, NRAS, HRAS, BRAF, and RASGAPs* in (**A**) lung adenocarcinoma (*n* = 230), (**B**) skin cutaneous melanoma (*n* = 287) and (**C**) colorectal adenocarcinoma (*n* = 212) from TCGA datasets are shown. Data were extracted through cBioPortal and presented as OncoPrint. Color coding indicates mutation type: red, homozygous amplification; blue, homozygous deletion; green, missense mutation; brown, inframe putative driver mutation; black, truncating mutation. Left, mutation percentage.

Corresponding with reported activity of NF1 as a tumor suppressor and negative regulator of RAS in lung adenocarcinomas [38], truncating mutations in *NF1* tend to be mutual exclusive with activating mutations in *RAS* and *BRAF* (TCGA) in these tumors (Figure 1A). Although the sample size of this lung adenocarcinoma cohort is too small to obtain reliable numbers for low abundant deletion and inactivating mutation frequencies in most other RASGAP genes, inactivating mutations in *RASA1* seem, like NF1, mutual exclusive with other activating mutations of the MAPK signaling pathway (Figure 1A).

The mutually exclusivity between loss-of-function mutations in *NF1* and oncogenic mutations in *RAS* and *BRAF* is also observed in melanoma patients (TCGA) (Figure 1B). However, a number of melanoma patients do have tumors that present both truncating mutations in *NF1* as well as oncogenic mutations in *BRAF*. Interestingly, all of the BRAF mutations that show co-occurrence with NF1 truncating mutations, both lung adenocarcinoma and melanoma samples, do not present the V600E hotspot mutation. Indeed, the non-V600E activating mutations in *BRAF* only induce weak oncogenic BRAF activity [49], suggesting that co-occurrence with NF1 loss, is required to obtain sufficient levels of RAS-ERK signaling. The frequency of inactivating alterations in the other RASGAP genes in this cohort of melanoma patients is again infrequent and too low to indicate their potential role in cancer development and progression (Figure 1B).

In contrast to lung adenocarcinoma and melanoma patients, the numbers of inactivating mutations in colorectal adenocarcinoma patients are low in all RASGAP genes (TCGA), including NF1 (Figure 1C). For CRC, low abundant mutation frequencies of RASGAPs might be the result of tissue-specific mechanisms of MAPK pathway activation and questions whether the loss of RASGAPs can actually play a substantial role in tumor progression of CRCs. Alternatively, other mechanisms affecting RASGAP protein levels, such as post-translational modifications affecting protein stability as well as gene silencing, can also account for decreased RASGAP activity, but this data is not present in sufficient quality and quantity to provide us more insight on functional mutually exclusivity [25, 40].

Thus, whereas mutual exclusivity of NF1-loss, in combination with previous experimental data [26, 28, 29, 36, 38], support NF1 as a strong oncogenic driver in lung adenocarcinoma and melanoma, the general oncogenic role of RASGAP deficiency in CRC remains inconclusive.

### A CRISPR-mediated RASGAP knock out screen identifies NF1 as the only RASGAP which depletion enables enhanced tumor growth and EGF-independent survival

**Figure 2:**
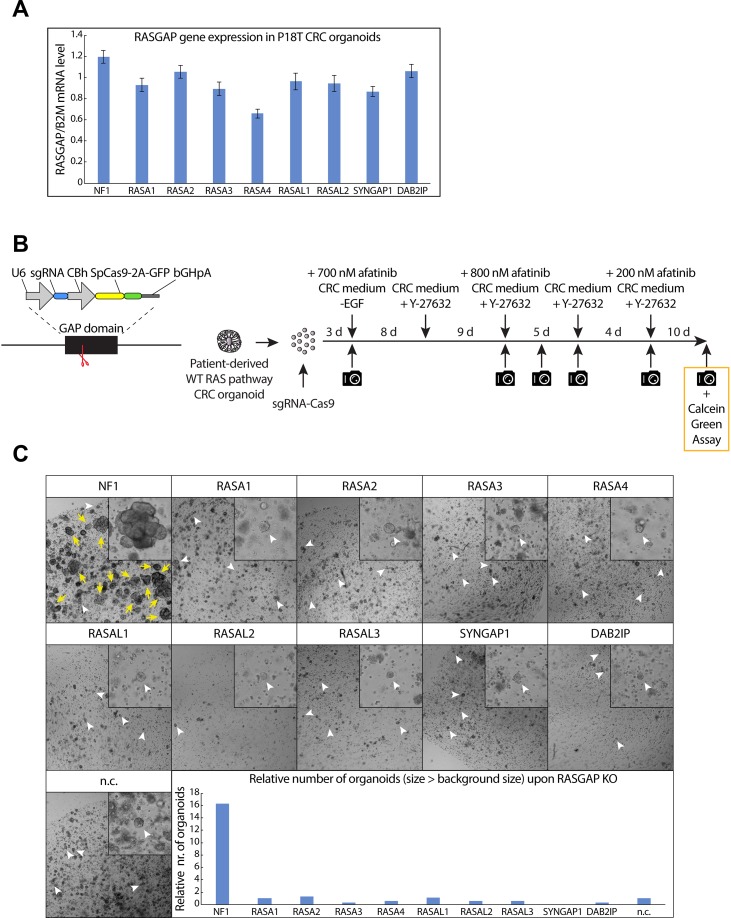
CRISPR screen against RASGAPs in patient-derived CRC organoids reveals increased growth and EGF-independent survival upon loss of NF1 GAP activity (**A**) The mRNA expression level of 9 RASGAPs containing an active GAP domain was analyzed in P18T organoids using qPCR. The relative expression of each RASGAP gene was normalized to the *B2M* housekeeping gene (representative from *n* = 3 independent experiments). (**B**) Left; schematic representation of expression plasmid containing both an U6 promoter-driven sgRNA and a CBh promoter-driven SpCas9-2A-GFP was used to target the RASGAP domain. Right; schematic overview of the RASGAP knock out screen in P18T patient-derived CRC organoids that are wild type for the RAS signaling pathway. (**C**) P18T CRC organoids in selection medium that have been transfected with indicated sgRNAs and Cas9. White arrow heads indicate representative background organoids. Yellow arrows indicate successful organoids that are significantly larger than background. Bar graph depicts the relative number of organoids with a size larger than background organoids as determined in the negative control. Area of alive RASGAP knock out organoids was measured using calcein green assay (see Materials and Methods).

To circumvent the lack of patient-data regarding loss of RASGAP expression, we set out to test the function of RASGAPs in CRC in an experimental setting. For this we utilize a patient-derived tumor organoid (P18T) with loss-of-function mutations in the WNT (APC) and TP53 pathway, but that is wild type for the RAS pathway and as such requires EGF-mediated growth factor signaling for growth and survival [50]. With the exception of RASAL3, which shows specific expression in the hematopoietic lineage in mice and humans (Supplementary Figure 1A), we identified the other RASGAPs at similar expression levels (Figure 2A) [51, 52]. To investigate whether loss of RASGAPs enables EGF-independent tumor cell growth and survival in CRCs, we depleted the activity of each RASGAP separately in P18T organoids using CRISPR-induced knock outs by targeting Cas9 cleavage activity against the conserved arginine finger in the catalytic GAP domain (Figure 2B) [53, 54]. The generation of knock outs was confirmed by DNA sequencing analysis of multiple monoclonal RASGAP knock out organoids (Supplementary Figure 1B). Subsequently, the population of RASGAP deficient CRC organoids were intermittently cultured in the absence of EGF signaling activity (EGF depleted growth medium containing afatinib, a small molecule inhibitor that targets the tyrosine kinase receptors EGFR, HER2 and HER4) (Figure 2B).

Surprisingly, we found that only the loss of NF1 GAP activity, but not of the other RASGAPs, resulted in a significant organoid growth upon intermittent EGFR inhibition (Figure 2C). In contrast to autonomous KRAS mutations, inactive NF1 did not result in complete EGF independence as organoid sizes remained small (but survived) upon continuous EGFR inhibition. However, elevated growth was observed under culture conditions with minimal EGFR stimulation (data not shown).

Since small sized organoids were observed for most conditions, including the negative control, we labelled all living organoids at the end point with calcein green to perform accurate measurements of number and size of organoids. Again, it illustrates that the number of organoids that are significantly larger in size is only observed after loss of NF1 activity in relation to the other RASGAP knock out organoids (Figure 2C and Supplementary Figure 1C). Importantly, to exclude the possibility that this observation was influenced by patient specific effects, we performed a similar experiment in which we targeted upstream exons or the GAP domains of *NF1* and *RASA1* in engineered tumor organoids that are also deficient in APC and TP53 (commonly referred to as tumor progression organoid 2 (TPO2)) [55]. Reassuring, a similar phenotype was observed in the TPO2 organoids, in which again only the loss of NF1 resulted in an increased outgrowth of large organoids as compared to control (Supplementary Figure 2A and 2B).

Together, these results indicate that only loss of NF1 activity promotes the outgrowth of CRCs upon limited EGFR signaling. The lack of participation by other RASGAPs is surprising, but consistent with the non-redundant and tissue-specific functions of RASGAPs [56].

### Generation of NF1 and RASA1 knock out organoid lines independent of phenotypic selection

**Figure 3:**
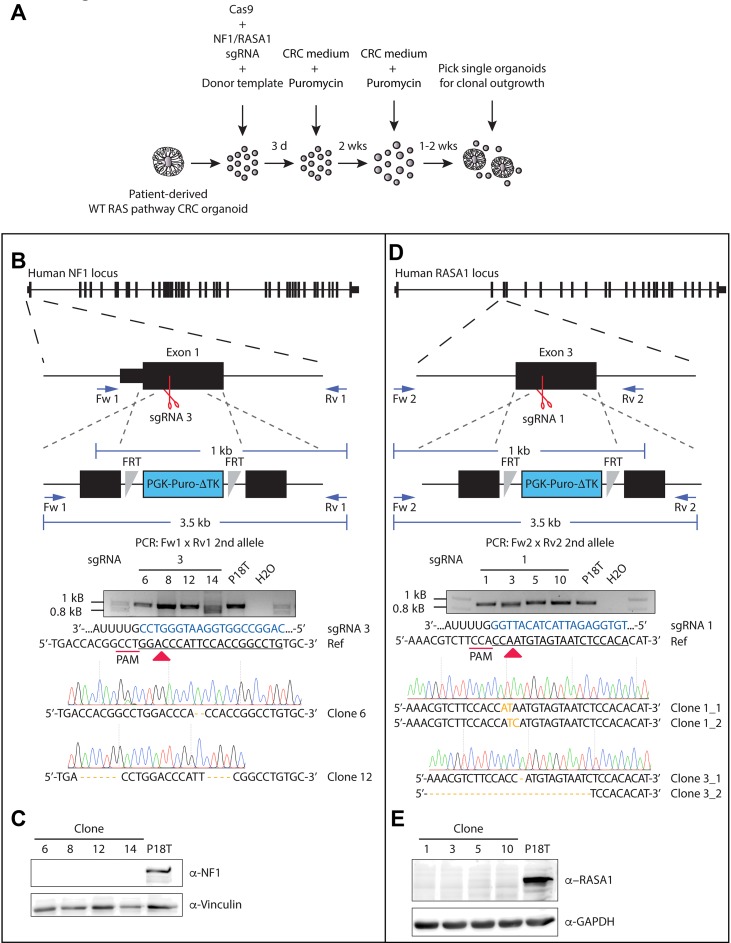
Generation of CRISPR-mediated NF1 and RASA1 knock out in patient-derived CRC organoids (**A**) Selection strategy to generate NF1 and RASA1 knock out organoids after CRISPR-mediated homologous recombination. (**B**, **D**) Genetic strategy to target the *NF1* and *RASA1* locus for homologous directed repair via the CRISPR/Cas9. The structure of the *NF1* and *RASA1* gene and the targeted exon is depicted at the top. Black boxes illustrate exons, separated by introns. Red scissors show sgRNA-generated double stranded breaks. Blue arrows illustrate PCR primer pairs. The agarose electrophoresis gel shows the ~1kb PCR product of the allele that was repaired by NHEJ of *NF1* (Clone # 6 and 12) and *RASA1* (Clone # 1 allele 1 and 2, Clone # 2 allele 1 and 2) in selected clones. Sanger sequencing indicate the introduced small indels per clone. Nonmatching bases are shown in orange. Regions of the sgRNA complementary to the protospacer (underlined) are shown in blue. Red arrow heads indicate cleavage sites. (**C**, **E**) Western blot analysis for NF1 and RASA1 presence in the indicated organoid lines.

As a tumor suppressive role has predominantly been suggested for both NF1 and RASA1 [25, 40], we continued to investigate both their function in CRC. Since the CRISPR/Cas9-induced truncating mutations in the GAP domain, which lies central in the protein sequence, may have resulted in the generation of dominant-negative versions, we also generated complete *NF1* and *RASA1* knock out lines in the CRC background of P18T (Supplementary Figures 3 and 4). Underscoring the role of NF1 deficiency in CRC progression upon EGFR signal inhibition, all outgrowth clones of NF1 were true knock outs (10/10), in contrast to only half of the RASA1 clones (7/14). Indeed, our selection method on phenotype could potentially result in the positive selection of undesired off-target mutations, which alone or together with NF1 or RASA1 inactivating mutations may lead to EGF independence and increased cell proliferation. Therefore, additional knock outs were generated without phenotypic selection, but by means of integration of puromycin selection cassettes via homologous recombination (Figure 3A) [57].

Indeed, this strategy led to the successful generation of non-functional *NF1* and *RASA1* genes independent of selection through EGFR inhibition (EGFRi) (Figure 3B and 3D). Of note, the puromycin selection cassette was properly integrated into the *NF1* gene body (Supplementary Figure 5A), accompanied by the introduction of small indel mutations in the ‘secondary’ allele (Figure 3B). Although the selection cassette was also integrated into the genome of *RASA1* knock out organoids (Supplementary Figure 5B), we could not confirm its exact integration site, probably due to its random genomic integration. Most importantly, we did confirm introduction of small indel mutations at both alleles of the *RASA1* gene (Figure 3D). Moreover, Western blot analysis of the different clones showed complete loss of NF1 and RASA1 protein in P18T CRC organoids (Figure 3C and 3E, and Supplementary Figure 5C). As we were able to generate multiple RASGAP knock out clones, we continued working with two different clones of each RASGAP knock out to exclude clonal effects.

### NF1 deficiency, but not RASA1, causes intrinsic EGF-independency

**Figure 4:**
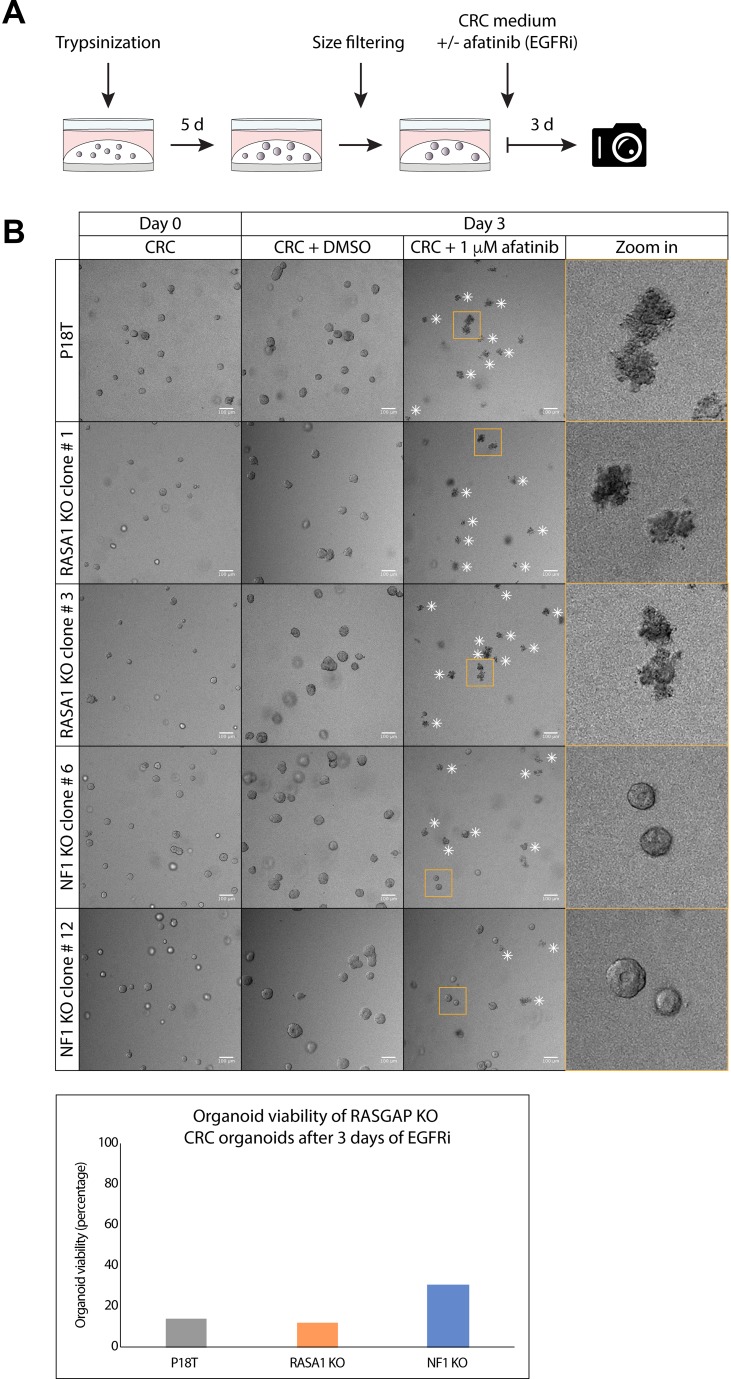
Puromycin selected NF1 knock out CRC organoids show insensitivity to EGFR inhibition (**A**) A schematic overview illustrating the strategy to score sensitivity of NF1 and RASA1 knock out organoids of similar size treated with colorectal cancer (CRC) medium containing either DMSO or 1 μM afatinib (EGFRi) for 72 hours. (**B**) Representative pictures of the parental patient-derived CRC organoids P18T and P18T RASA1 (clone # 1 and # 3) or NF1 (# 6 and # 12) knock out organoids prior (day 0) and after 72 hours of DMSO or 1 μM afatinib treatment (Day 3). White asterisks indicate dead organoids. Scale bars, 100 μM. Bar graph depicts the percentage of living organoids (out of 100 organoid counts) based on morphology.

First, we validated whether loss of NF1, but not RASA1, induced intrinsic EGF independence for CRC organoid survival by examining the effect of afatinib (EGFR/HERi) treatment on organoid viability by microscopy. Therefore, 5 days after trypsinization the parental P18T organoids, as well as the NF1 and RASA1 knock out organoid cultures, were filtered to homogenize their size and subsequently cultured with afatinib or DMSO (control) for 72 hours (Figure 4A). In agreement with our RASGAP knock out screen (Figure 2), complete loss of NF1 indeed resulted in a slight increase in EGF independence in terms of cell survival, showing healthy but small organoids after EGFRi treatment. In contrast, the parental P18T, as well as the RASA1 knock out lines, predominantly died in the presence of EGFR inhibition (Figure 4B).

### Loss of NF1 expression enhances basal RAS-ERK activity in CRC organoids

For both NF1 and RASA1 it has been demonstrated that they can affect RAS and ERK activity in various cell lines [35, 38, 45]. To investigate the molecular mechanism that underlies increased tumor growth and EGF-independent survival upon loss of NF1 expression, we analyzed the activity of the RAS-MAPK signaling pathway.

**Figure 5:**
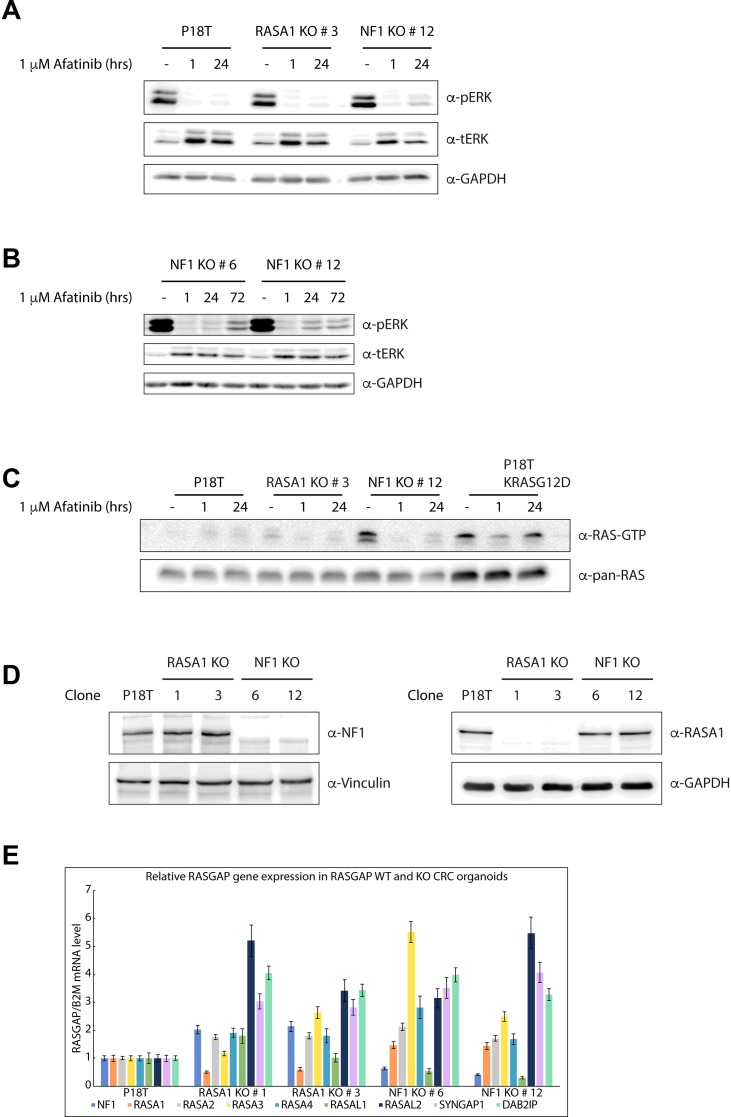
Puromycin selected NF1 knock out CRC organoids show enhanced RAS and ERK activation (**A**) In comparison to P18T and P18T RASA1^KO^ (clone # 3), predominantly P18T NF1^KO^ (clone # 12) organoids show enhanced basal and reactivated ERK phosphorylation levels after 24 hr treatment with CRC medium containing 1 μM afatinib. Representative from *n* = 3 independent experiments. (**B**) NF1-deficient organoids (clone # 6 and # 12) show residual ERK phosphorylation after treatment with CRC medium containing 1 μM afatinib with varying kinetics. (**C**) Loss of NF1 (clone # 12) leads to elevated levels of RAS activity (GTP-loading) at basal conditions compared to P18T and P18T RASA1^KO^ (clone # 3) CRC organoids. The presence of an oncogenic mutation in KRAS (P18T KRAS^G12D^) leads to elevated and sustained high levels of RAS activity (GTP-loading) at basal and in afatinib-treated conditions, respectively. RAS immunoblots from RAS pull-down assays are shown (RAS-GTP), together with a RAS immunoblot from total cell lysates as loading control. HRAS, KRAS, and NRAS isoforms are detected. Representative from *n* = 2 independent experiments. (**D**) Immunoblots of P18T, P18T RASA1^KO^ (clone # 1 and 3), P18T NF1^KO^ (clone # 6 and 12) CRC organoids indicate that the loss of RASA1 does not result in elevated protein levels of NF1, and vice versa. Representative from *n* = 3 independent experiments. (**E**) The relative expression levels of indicated RASGAPs genes that contain an active GAP domain were analyzed in P18T, P18T RASA1^KO^ (clone # 1 and 3), P18T NF1^KO^ (clone # 6 and 12) CRC organoids using RT-PCR. The relative expression of each RASGAP gene was normalized to the *B2M* housekeeping gene (representative from *n* = 3 independent experiments).

Consistent with the phenotypes of our RASGAP knock out lines, only the loss of NF1 expression in CRC organoids resulted in enhanced ERK activation under basal conditions (Figure 5A and Supplementary Figure 6A), which was further verified by quantification (Supplementary Figure 7A).

Whereas inhibition of EGF signaling clearly reduced the levels of active (phosphorylated) ERK in all three organoid types (i.e. P18T, NF1 and RASA1 KOs) after 1 hour of afatinib treatment, a substantial reactivation of ERK was observed in alive NF1 knock out organoids after 72 hours of afatinib treatment (Figure 5A and 5B). Importantly, similar reactivation effects albeit with different kinetics were observed in different NF1 KO clones (Figure 5B and Supplementary Figure 6A), explaining their enhanced survival upon EGFR inhibition.

Since the predicted function of NF1 GAP activity is to enhance the intrinsic GTP hydrolysis rate of GTP-bound RAS, we examined the amounts of active, GTP-bound RAS that are present in the absence of NF1. As a positive control, we also analyzed RAS-GTP levels in a P18T organoid line in which an activating mutation in KRAS (G12D) was introduced by CRISPR technology [58]. In comparison with the parental P18T line, loss of NF1 clearly enhanced RAS-GTP levels at basal conditions to similar levels as observed in KRAS mutant P18T organoids. However, whereas a substantial fraction of GTP-bound RAS was detected in oncogenic mutant KRAS organoids after 24 hours of EGFR inhibition, this was not observed in NF1 knock out organoids (Figure 5C, Supplementary Figures 6B, and 7B). This discrepancy on RAS-GTP loading may very well be explained by the fact that NF1, in contrast to self-autonomous oncogenic KRAS, acts as an amplifier of RAS-mediated signaling. Therefore, the effect of NF1-loss can only manifest itself in the presence of RAS activating signals, i.e. in the presence of incoming EGF signaling. Indeed, in the RASGAP CRISPR screen we observed that the largest differences in organoid growth and viability were obtained in the presence of minimal EGF signaling.

As expected by the sensitivity of RASA1 knock out organoids for EGFR signal inhibition, loss of RASA1 did not enhance or sustain RAS-MAPK signaling in case of inhibition, nor under normal growth conditions (Figure 5C, Supplementary Figures 6A, 6B and 7A, 7B). Subsequently, we analyzed protein levels of both NF1 and RASA1 in the RASA1 knock out organoid lines to investigate whether upregulated expression of NF1 acts as a compensation mechanism, but we did not observe any differences in the knock-out lines as compared to wild type P18T (Figure 5D). To explore whether other RASGAPs compensate for the loss of RASA1 activity, we examined the mRNA expression levels of the other RASGAPs in *RASA1* and *NF1* knock out organoids. In addition to some clonal variability, we detected slight increased expression for most RASGAP genes in the case of NF1 or RASA1 deficiency (Figure 5E). However, no clear candidates could be identified which expression level is suggestive for a redundant function to RASA1 deficiency and might have explained a compensatory mechanism that prevents aberrant RAS activation.

Intriguing, while it has been demonstrated that all RASGAPs contain an active GAP domain, our data only identifies NF1 as a bona fide amplifier of RAS-mediated MAPK signaling in patient-derived CRC organoids.

### NF1-deficient CRC organoids show enhanced organoid survival and growth upon release of RAS-MAPK pathway inhibition

A recent study showed that NF1 mutations correlated with a poor response to cetuximab-based EGFR inhibition and decreased progression free survival of mCRC patients [46]. Resistance to targeted therapy is often the result of residual RAS-ERK signaling activity [59]. In agreement, we detected enhanced organoid survival and residual ERK activity in NF1-deficient CRC organoids upon long-term EGFR inhibition with afatinib. Therefore, as NF1 loss predominantly amplifies RAS-MAPK signaling activity in unperturbed conditions, we hypothesized that the phenotype of NF1 deficiency might manifest itself most evidently upon the release of EGFR inhibition. In contrast, desired cytotoxicity upon targeted therapy should be achieved upon full RAS-MAPK signal inhibition.

**Figure 6:**
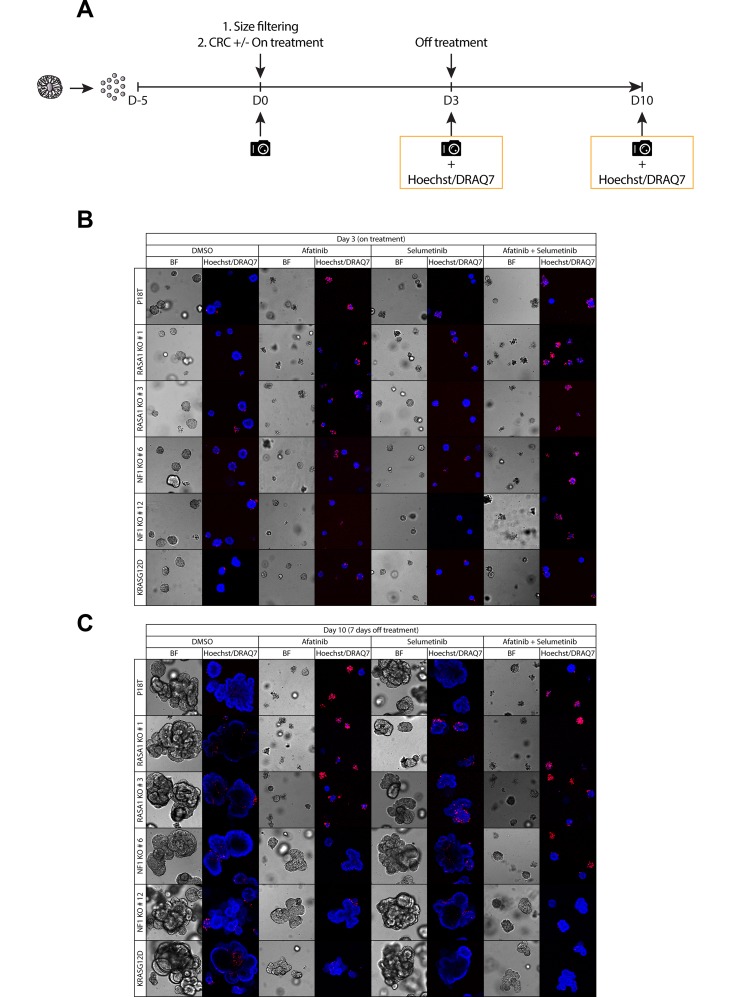
Puromycin selected NF1 knock out CRC organoids show enhanced organoid growth upon release of RAS-MAPK pathway inhibition (**A**) A schematic overview illustrating the strategy to score sensitivity and outgrowth of P18T parental, RASGAP knock out, and oncogenic mutant KRAS organoids of similar size during and after treatment with colorectal cancer (CRC) medium containing either DMSO, 1 μM afatinib (EGFRi), 1 μM selumetinib (MEKi), or a combination of 1 μM afatinib and 1 μM selumetinib. Organoid size and frequency of alive organoids was quantified after 72 hr of drug treatment and after 7 days of drug withdrawal by phenotypic analysis. (**B**) Representative zoom-in pictures of the parental patient-derived CRC organoids P18T KRAS^WT^, KRAS^G12D^, and P18T RASA1 (clone # 1 and # 3) or NF1 (# 6 and # 12) after 72 hours of DMSO or targeted drug treatment (on treatment). (**C**) Representative zoom-in pictures of the parental patient-derived CRC organoids P18T KRAS^WT^, KRAS^G12D^, and P18T RASA1 (clone # 1 and # 3) or NF1 (# 6 and # 12) after 7 days of DMSO or drug withdrawal (off treatment). Hoechst and DRAQ7 was used to visualize nuclei and dead cells, respectively.

To investigate this, we set up a drug screen to measure the phenotypic response of parental P18T, both RASGAP knock outs, as well as KRAS mutant P18T organoids, during and after mono- and combinatorial targeted therapies against the RAS-MAPK signaling pathway (Figure 6A).

In line with our previous results, NF1 knock out organoids show some resistance to EGFR-targeted therapy (afatinib) in comparison to wild type organoids, but not as evident as the resistance observed in KRAS mutant organoids (Figure 6B, Supplementary Figure 8A, 8B). Whereas the observed effects remain subtle for the monotherapies, combined inhibition of MEK (selumetinib) and EGFR/HER (afatinib) resulted in an improved cytotoxic response in NF1 knock out organoids (Figure 6B, Supplementary Figure 8A, 8B).

Most striking however, when organoids were released from targeted inhibition, a tremendous organoid outgrowth was observed in NF1-deficient organoids that was only matched by KRAS mutant organoids. In contrast, parental KRAS^WT^ and RASA1 knock out organoids remained small even upon drug withdrawal (Figure 6C and Supplementary Figure 8). In contrast to the autonomous KRAS^G12D^ mutation, the phenotype of NF1 deficiency manifests itself predominantly under challenging EGFR signaling conditions, but is not able to rescue complete inhibition of the MAPK signaling pathway. These result are consistent with observations in an *in vitro* and *in vivo* model of NF1-deficient lung adenocarcinoma treated with EGFR and MEK inhibitors [38]. Translating these results to a clinic setting suggests that NF1-deficient mCRC are able to show favorable responses towards targeted inhibition of the RAS-MAPK pathway, but only under strict full inhibitory conditions using combinatorial targeting strategies with ideally a continuous treatment regime.

## DISCUSSION

Data analysis of patients with lung adenocarcinoma and melanoma show that of all RASGAPs only inactivating mutations in *NF1* tend to occur in a mutually exclusive manner with activating hotspot mutations in *KRAS*, *NRAS* and *BRAF*. This suggests that the loss of NF1 is sufficient to drive aberrant activity of the RAS-MAPK signaling pathway in the absence of other mutations in the RAS signaling pathway. However, the data is less clear for CRCs, in which the frequency of loss-of-function mutations in RASGAPs are very low, bringing into question whether the loss of RASGAPs plays an important role in CRC development and progression. Moreover, the identification of RASGAPs in large GWAS studies and RASGAP expression analysis studies has led to their family-wide association with tumorigenesis [39, 47, 48, 60–65], in contrast to mutation frequencies that mainly point to NF1 [25].

To end the debate, we set out to investigate the functional relationship between all RASGAPs and tumor growth in the presence and absence of EGFR signaling in colorectal tumors. Therefore, we performed a CRISPR-mediated knock out screen to study RASGAPs with a functional GAP domain in patient-derived CRC organoids. Surprisingly, of all the potential RASGAPs, we identified that only the loss of NF1 resulted in increased tumor growth and EGF-independent cell survival. Importantly, our observations were made in multiple genetic backgrounds and by multiple genetic strategies. On the biochemical level, loss of NF1 results in enhanced RAS-ERK activation but does require presence of active EGFR signaling to do so. Indeed, in contrast to autonomous KRAS^G12D^, NF1 loss mainly acts as a signal amplifier. As such, NF1 deficient tumors remain vulnerable to targeted inhibition of the MAPK pathway, but only under complete inhibitory conditions. Under normal growth conditions, i.e. presence of EGF signals, NF1 deficiency might be responsible for aberrant MAPK signal activation in CRCs that are wild type for known MAPK driver genes.

Several studies have shown that the expression of NF1 is altered in a number of sporadic cancers [25, 40]. Moreover, loss of NF1 expression has been observed in lung adenocarcinomas and melanomas that are resistant to treatment with EGFR [38] and BRAF [28, 29, 36, 37] inhibitors, respectively. Whereas reports on the frequency of genetic alterations of NF1 in CRC show varying results, a recent study associated mutant NF1 with tumor progression and anti-EGFR therapy resistance [46]. To our knowledge we are the first to present direct loss-of-function data that of all RASGAPs only the loss of NF1 promotes enhanced tumor growth and EGF-independent survival in CRC. Next generation sequencing studies identified that the NF1 gene is altered in approximately 5-6% of colorectal carcinomas [66]. Since loss of NF1 can also be accomplished via mechanisms other than loss-of-function mutations, such as epigenetic gene silencing or aberrant protein stability [33, 67], analysis of NF1 protein levels in tumor biopsies may be of importance to correctly stratify the patient population for targeted therapy against the MAPK signaling pathway.

Although enhancing RAS-ERK signaling during normal tumor growth conditions, NF1 deficiencies are not as dominant drivers of MAPK signaling activity as oncogenic versions of KRAS. As a result, patients with NF1 mutant CRCs are much more likely to respond to combinatorial targeting of the MAPK signaling pathway than RAS mutants in case full inhibition is achieved. In relation to mutational co-occurrence, it is conceivable that loss of NF1 can enhance otherwise weak activating mutations of the MAPK signaling pathway to reach optimal levels of pathway activation for efficient tumor growth [68].

To confirm the role of NF1 as an amplifier of the RAS effector, we also tried to determine the levels of RAS-ERK activation upon minimal perturbation of EGFR signaling with low concentrations of afatinib. In contrast to P18T and RASA1 knock outs, enhanced levels of active RAS-ERK signaling, comparable to levels of KRAS mutants, was again observed at basal conditions in NF1 knock out organoids. However, elevated levels of active GTP-bound RAS proteins were challenging to detect under minimal perturbation of EGFR signaling, among others due to drug titration difficulties to achieve reduced but remaining EGFR activity, as well as different distribution of RAS proteins at the membrane under changing EGFR stimulation that can mask the effect of NF1 loss on the membrane pool [69].

Whereas several publications have proposed a tumor suppressor role for RASA1 in CRC [43–45], we did not observe elevated levels of RAS and ERK activity in RASA1 knock out organoids at basal conditions. Importantly, loss of RASA1 was not sufficient to promote EGF-independent survival. Moreover, the upregulated expression levels of other RASGAPs did not point to a clear candidate that could compensate for its loss. From a molecular perspective, it is not clear whether RASA1 is indeed a negative regulator of RAS in colon, or that the existing pool of NF1 or other RASGAPs is sufficient to compensate its absence. For instance, differences in the RAS binding groove have previously been identified between RASA1 and NF1, which have been attributed to higher RAS binding affinities for NF1 [53, 54]. Moreover, other studies have demonstrated that RASA1 may have increased activity toward R-RAS compared to other RAS proteins, thereby affecting RalA activity, but not PI3K or MAPK signaling [70, 71]. Our data, together with observations that ubiquitous loss of RASA1 in mice does not lead to spontaneous tumor formation, questions whether RASA1 functions as an essential tumor suppressor in the gut [72].

Together, using patient-derived CRC organoids, we reveal that of all RASGAPs with functional GAP domains, only NF1 deficiency promote cell survival and enhanced tumor growth upon challenging EGF signaling conditions in human CRC samples. On the basis of our data, we propose that NF1 protein levels should be determined in CRCs prior initiation of targeted therapy against the MAPK pathway. Patients with NF1-deficient tumors are likely to be unresponsive to anti-EGFR targeted monotherapy. However, on a positive note, we did observe that these tumors, unlike KRAS mutant colorectal tumors, are vulnerable towards combinatorial targeting strategies against the RAS-MAPK pathway.

## MATERIALS AND METHODS

### Patient-derived organoid culture and maintenance

The patient-derived organoids derived in this study were previously established and characterized (van de Wetering *et al*., 2015 and Drost *et al*., 2015). Human CRC and TPO2 (APC-/-, TP53-/-) colon organoids were cultured as described previously (van de Wetering *et al*., 2015, Drost *et al*., 2015, Verissimo *et al*., 2016). Culture medium containen advanced DMEM/F12 medium (Invitrogen) with 1% Penicillin/Streptomycin (P/S, Lonza), 1% Hepes buffer (Invitrogen) and 1% Glutamax (Invitrogen), 20% R-spondin conditioned medium, 10% Noggin conditioned medium, 1x B27 (Invitrogen), 1.25 mM n-Acetyl Cysteine (Sigma-Aldrich), 10 mM Nicotinamide (Sigma-Aldrich), 50 ng/ml EGF (Invitrogen), 500 nM A83-01 (Tocris), 10 μM SB202190 (ApexBio) and 100 μg/ml Primorcin (Invitrogen). Organoids were splitted through Trypsin-EDTA (Sigma-Aldrich) treatment. Culture medium after splitting was supplemented with 10 μM Y-27632 dihydrochloride. For selection of RASGAP knock out mutants, organoids were grown in culture medium containing 1–2 μM puromycin or lacking EGF and containing 0.2–1.0 μM of afatinib (Selleck Chemicals).

The Jurkat cells were cultured in RPMI 1640 medium supplemented with 10% FBS, 1% Penicillin/Streptomycin (P/S, Lonza) and 1% Glutamax (Invitrogen).

### Organoid transfection and genotyping

The transfection protocol of P18T and TPO2 organoids was previously described in detail by Fujii *et al.* (2015). Three days after transfection, culture media plus Y-27632 was exchanged with selection medium. After puromycin selection, surviving clones were picked and subjected to genotyping to detect the presence of insertions and deletions.

For genotyping, genomic DNA was isolated using Viagen Direct PCR (Viagen). The presence of insertions or deletions in RASGAPs was verified by using the PCR product obtained using the following primers:

NF1_fw 5′-GACCCTCTCCTTGCCTCTTC-3′, NF1_rv 5′-GGTGGCTCTGAAGCAGTTTC-3′, RASA1_fw1 5′-GACTCTTCCTTTTCCTCCCG-3′, RASA1_rv1 5′-G CAGTTTGGTGAGAGCCATG-3′, RASA1_fw2 5′-GT TGGGCATTACTGTGCTG-3′, RASA1_rv2 5′- GGTG GTGCAACTGGGTAAAG-3′, NF1_fw GAP domain 5′-GTACACTGTTAAATCTCAGG-3′, NF1_rv GAP domain 5′-AGAGGATGTGATCACAATTC-3′, RASA1_fw GAP domain 5′-GTCTTACAGAGTTAAGTCTG-3′,

RASA1_rv GAP domain 5′-GTATTACAGACAGGTGT AAC-3′, RASA2_fw GAP domain 5′-CTTATGCCTTCT AGTATGTC-3′, RASA2_rv GAP domain 5′-TGCTTCTA AAGTGTTCAGTC-3′, RASA3_fw GAP domain 5′-GT GTTGACACAGGACGGTTC-3′, RASA3_rv GAP domain 5′-GGAGTACACAGGGAACATCC-3′,

RASA4_fw GAP domain 5′-AGAACACTGGGAGGTG TTTG-3′, RASA4_rv GAP domain 5′-CGAACTCCTG ACCTTAAGTG-3′, RASAL1_fw GAP domain 5′-TGTG CACCTCCAGACAGTTG-3′, RASAL1_rv GAP domain 5′-GACCATGCAGGAAGAGGTTC-3′, RASAL2_fw GAP domain 5′-CAGCATTTCCAGGATG TCTG-3′, RASAL2_rv GAP domain 5′-AGCAGTGTA TGCTGACAAGG-3′, RASAL3_fw GAP domain 5′-GC CTAAGCATCAGCTACAAG-3′, RASAL3_rv GAP domain 5′-GTCTTCAGGTTATTCCGGAG-3′,

SYNGAP1_fw GAP domain 5′-CACATCCTGCAGAGT ACAGG-3′, SYNGAP1_rv GAP domain 5′-ACAAGAG GGTGTGGTCACAC-3′, DAB2IP_fw GAP domain 5′-C ACCAGTTCTAGGCTCCTAC-3′, DAB2IP_rv GAP domain 5′-ACTTGCTGGGATCCACTTCG-3′.

Products were sequenced using the following primers:

NF1 exon 1 5′-CTTCCTTTCCTCCAGAGCCTG-3′,

RASA1 exon 1 5′-CACAAGCTGCCCTCTCCCTT-3′,

RASA1 exon 3 5′-CAAATAAACTTTGAGTGGTA-3′,

NF1 GAP domain 5′-GTACACTGTTAAATCTCAGG-3′,

RASA1 GAP domain 5′-GTACTTTCAACGCTGCAC-3′, RASA2 GAP domain 5′-CCTTCCCATCAATAGATC-3′,

RASA3 GAP domain 5′-GTGTTGACACAGGACGGT TC-3′, RASA4 GAP domain 5′-GAACACTGGAGTCGA AGTC-3′, RASAL1 GAP domain 5′-CTGGAAGAATC ATGACTCC-3′, RASAL2 GAP domain 5′-CCAGTGCG TCATGAAGATAC-3′, RASAL3 GAP domain 5′-GTCT TCAGGTTATTCCGGAG-3′, SYNGAP1 GAP domain 5′CACGAGATTGGGTTGTGC-3′, DAB2IP GAP domain 5′-CTAGGTCTGGAATCCTAG-3′.

In addition, the CloneJET PCR Cloning Kit was used to confirm indel generation in NF1 knock outs #1 and #6 and of RASA1 knock outs #1 and #3.

The presence of the puromycin selection cassette was verified by using the PCR product obtained using primers:

Puro_1_fw 5′-GACCCTCTCCTTGCCTCTTC-3′,

Puro_1_rv 5′-GTTGGCGCCTACCGGTGG-3′,

Puro_2_fw 5′-ATGGGGACCGAGTACAAGCC-3′,

Puro_2_rv 5′-GTCGAAGATGAGGGTGAG-3′.

### Vector construction

The CRISPR guide RNA (sgRNAs) were designed by an online CRISPR design tool (http://crispr.mit.edu). The sgRNA guide sequences used can be found in the Supplementary Materials (Supplementary Table 1). The sgRNAs used for the RASGAP knock out screen were cloned into a plasmid (px458) expressing both sgRNA and hCas9-2A-GFP as previously described (Ran *et al*., 2013).

For CRISPR-mediated homologous recombination the human codon-optimized Cas9 expression plasmid was obtained from Addgene (41815). The sgRNA-GFP plasmid was obtained from Addgene (41819) and used as a template for generating target specific sgRNAs as described in detail by Drost *et al*. (2015). For the generation of the donor template, genomic DNA from P18T organoids was used to PCR amplify the NF1 and RASA1 homology arms using high-fidelity Phusion Polymerase (New England BioLabs). The 5′ homology arm of RASA1 spans the region Chr5:87331757-87332526, and the 3′ homology arm spans the region Chr5:87332575-87333379. The 5′ homology arm of NF1 spans the region Chr17:31094574-31095323, and the 3′ homology arm spans the region Chr17:31095361-31096136. The homology arms were cloned into a pBlueScript plasmid expressing a 3229-bp AATPB:PGKpuroDtk selection cassette (Schwank *et al*., 2013).

### Western blot assay and RAS-GTP pull down

Prior to cell lysis, organoids were incubated with 1 mg/ml dispase II (Invitrogen) for 10 minutes at 37° C to digest the BME. Western blot samples for NF1 and RASA1 protein levels were lysed using NETN buffer (50 mM Tris-HCL pH 8.0, 250 mM NaCl, 5 mM EDTA, 0.5% NP-40) containing Complete protease inhibitors (Roche). Western blot samples for phosphorylated ERK were lysed using RIPA buffer (50 mM Tris-HCL pH 8.0, 150 mM NaCl, 0.1% SDS, 0.5% Na-Deoxycholate, 1% NP-40) containing Complete protease inhibitors (Roche). Protein content was quantified using a BCA protein assay kit (Pierce™) and analyzed by Western blotting. Membranes were blocked and probed with antibodies directed against NF1 (RRID:AB_2149790), RASA1 (RRID:AB_303418), Vinculin RRID:AB_477629, a-tubulin (RRID:AB_477579), GAPDH (RRID:AB_2107445), pERK (RRID:AB_331646), and ERK (RRID:AB_390779).

Samples for RAS-GTP isolation were lysed using Ral lysis buffer (50 mM Tris-HCL pH 7.5, 200 mM NaCl, 2 mM MgCl2, 10% glycerol, 1% NP-40) containing Complete protease inhibitors (Roche). Lysates were normalized for protein levels using a BCA protein assay kit (Pierce™) and subsequently GTP-bound RAS was isolated via immunoprecipitation using recombinant RAS binding domain of RAF1 (RAF1-RBD). Protein lysates were run on SDS-PAGE gels and transferred to PVDF membranes (Millipore). Membranes were blocked and probed with antibodies directed against RAS (RRID:AB_397425). Organoid treatments: afatinib (Selleck Chemicals) 1 μM, 1 h and 24 h or DMSO.

### RNA isolation, cDNA preparation and qRT–PCR

Organoids were harvested in RLT lysis buffer and RNA was isolated using the Qiagen RNeasy kit (Qiagen) according to the manufacturer's instructions. Extracted RNA was used as a template for cDNA production using iScript™ cDNA Synthesis Kit (Bio-Rad) according to the manufacturer's protocol. qRT–PCR was performed using FastStart Universal SYBR Green Master mix (Roche) according to the manufacturer's protocol. Results were calculated by using the relative standard curve method. Primer sequences:

B2M_fw 5′-GAGGCTATCCAGCGTACTCCA-3′,

B2M_rv 5′-CGGCAGGCATACTCATCTTTT-3′,

NF1_fw 5′-GGATCCTACCAGGTTAGAACCATC-3′,

NF1_rv 5′-AGCTTTATTCAGTAGGGAGTGGC-3′,

RASA1_fw 5′-AATGCAGGATCAAGAACAAG-3′,

RASA1_rv 5′-AAGGCATCCTTTGTTTTACG-3′,

RASA2_fw 5′-GACTTGTGTAATCACAGTGG-3′,

RASA2_rv 5′-TACCCTGAACCTCTGAATTG-3′,

RASA3_fw 5′-AAGAGTGTTGAGCAGCCCAT-3′,

RASA3_rv 5′TAGAGAGGCTGGTCCCCTTTG-3′,

RASA4_fw 5′-CAGCCGGGACGACGTTATC-3′,

RASA4_rv 5′-CCACCCGCTGAAACCCTTAG-3′,

RASAL1_fw 5′-CGTGCTGGATGAGGACACTG-3′,

RASAL1_rv 5′-TCCCTGCTCAGCGAGATCTT3′,

RASAL2_fw 5′-CCCAACTCCATGGACACTGC-3′,

RASAL2_rv 5′-GGATGGAAGCCGAAAGCTCG-3′,

RASAL3_1_fw 5′-GGATCCAGATCGGATGCCTG-3′,

RASAL3_1_rv 5′-TCCCTAGAGCCCAGAGCAC-3′,

RASAL3_2_fw 5′-AACAGAACCGGAGACTGCTG-3′,

RASAL3_2_rv 5′-GCTCCAACCTGGCCTTTTTC-3′,

RASAL3_3_fw 5′-GCTCAAGAGGCTGAAAGAG-3′,

RASAL3_3_rv 5′-CAGGTCCAGTTCAGAGAGTG-3′,

DAB2IP_fw 5′-CATCATCAGCAGGTTGATGTCC-3′,

DAB2IP_rv 5′-AGCGGGCTTTTGTTTCTAATGC-3′,

SYNGAP1_fw 5′-ATGCAAAGCTTTAAGGAGTC-3′,

SYNGAP1_rv 5′-GTTCCTGATGAAGTTGTTACC-3′

### Targeted inhibitors

Afatinib and Selumetinib, were purchased from Selleck Chemicals. These compounds were dissolved in dimethylsulfoxide (DMSO, Sigma-Aldrich) and stored as 10 mM aliquots.

### Phenotypic drug screen and calcein green assay

Five days after organoid trypsinization, 1 mg/ml dispase II (Invitrogen) was added to the medium of the organoids and these were incubated for 15 min at 37° C to digest the BME. Subsequently, organoids were mechanically dissociated by pipetting, filtrated using a 40 μm nylon cell strainer (Falcon), resuspended in 75% BME/growth medium (40 organoids/μl) prior plating of two 10 μl drops on Nunc™ Lab-Tek™ II Chamber Slide™ Systems. After plating culture medium containing either 1 μM of afatinib, 1 μM of selumetinib, a combination of 1 μM of afatinib and 1 μM of selumetinib or DMSO was added. The labtek plates were mounted on an inverted confocal laser scanning microscope (Leica SP8X) and imaged using a 10X objective. For visualization of cell viability, organoids were incubated with 16.2 μΜ Hoechst 33342 (Life Technologies) and 1.5 μM DRAQ7™ (Cell Signaling #7406) for 30 min at 37° C prior imaging.

For the GAP domain knock out CRISPR screen, organoids were imaged by an inverted routine microscope (Nikon Eclipse TS100) using a 4X or 10X objective. For calculating organoid count and size, organoids were incubated for 45 minutes with 500 ml culture medium containing 5 μM calcein-green (Invitrogen). For the quantification of the organoid size and count, FIJI analysis software was used.

## SUPPLEMENTARY MATERIALS FIGURES AND TABLES


