# Supplementary material for: CRISPR-induced RASGAP deficiencies in colorectal cancer organoids reveal that only loss of NF1 promotes resistance to EGFR inhibition
Source: Oncotarget. 2019 Feb 15;10(14):1440–57. doi: 10.18632/oncotarget.26677 (PMC6402720; doi:10.18632/oncotarget.26677)
Supplement: Supplementary file 1 [file oncotarget-10-1440-s001.pdf]

CRISPR-induced RASGAP deficiencies in colorectal cancer organoids reveal that only loss of NF1 promotes resistance to EGFR inhibition

SUPPLEMENTARY MATERIALS

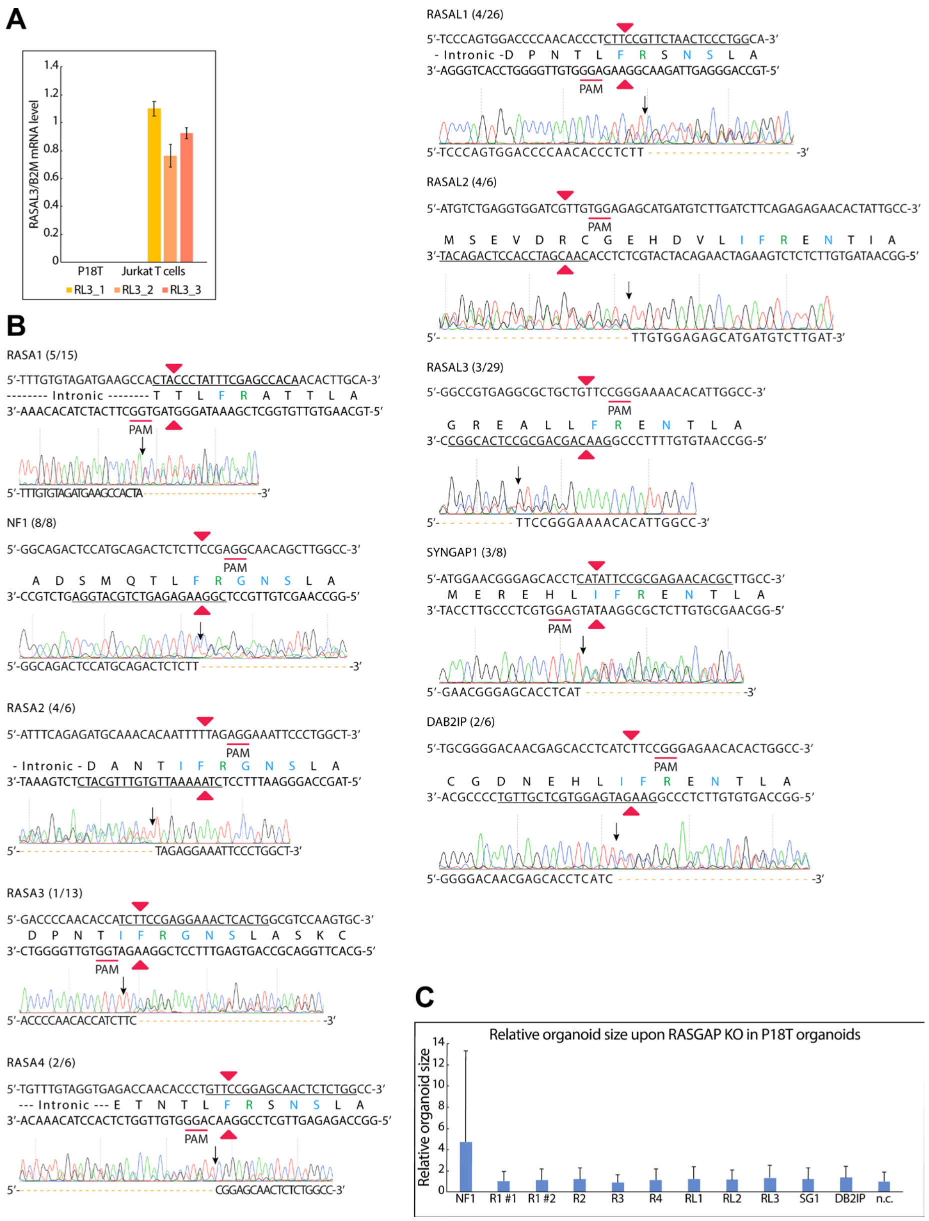

Supplementary Figure 1: CRISPR screen targeting GAP domains reveals increased cell growth upon NF1 loss in patient-derived CRC organoids. (**A**) The mRNA expression level of RASAL3 was analyzed in P18T organoids and Jurkat T cells using qPCR. The relative expression of RASAL3 was normalized to the *B2M* housekeeping gene (representative from $n = 3$ independent experiments). (**B**) Sanger sequencing results (representative of multiple monoclonal organoid lines) indicate the introduction of indels in the GAP domain of each RASGAP targeted by the CRISPR machinery. The number of mutant monoclonal organoids per total number of clones sequenced are indicated in brackets. Nonmatching bases are shown in orange. The protospacer is underlined. The catalytic arginine of the finger loop is depicted in green and the conserved residues in blue. Red and black arrow heads indicate cleavage sites. (**C**) The bar graph depicts the relative size of organoids (size > background) upon CRISPR-mediated knock out of indicated RASGAPs in P18T CRC organoids after selection with EGF depletion and afatinib treatment. Organoids size was measured by the calcein green assay (see Materials and Methods).

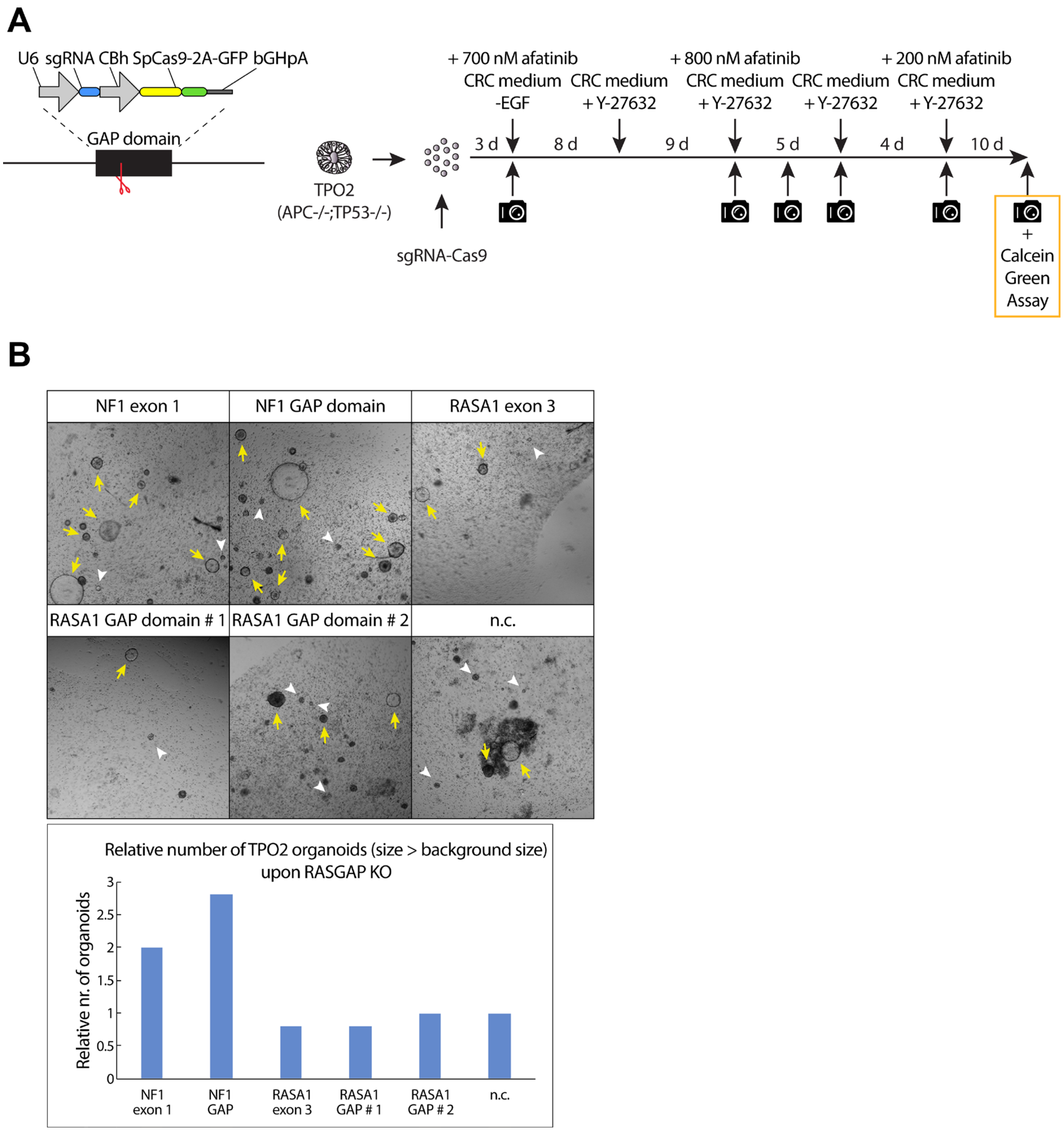

Supplementary Figure 2: CRISPR screen targeting GAP domains reveals increased growth and EGF-independent survival upon NF1 loss in human tumor progression organoids. (**A**) A schematic overview of the RASGAP knock out screen in engineered human colorectal tumor organoids, so-called TPO2 ($APC^{-/-}$, $TP53^{-/-}$). An expression plasmid containing both an U6 promoter-driven sgRNA and a CBh promoter-driven SpCas9-2A-GFP was used to target the RASGAP domain. (**B**) TPO2 organoids in selection medium that have been transfected with indicated sgRNAs and Cas9. White arrow heads indicate representative background organoids. Yellow arrows indicate successful organoids that are significantly larger than background. Bar graph depicts the relative number of organoids with a size larger than background organoids as determined in the negative control. Area of alive RASGAP knock out organoids was measured using calcein green assay (see Materials and Methods).

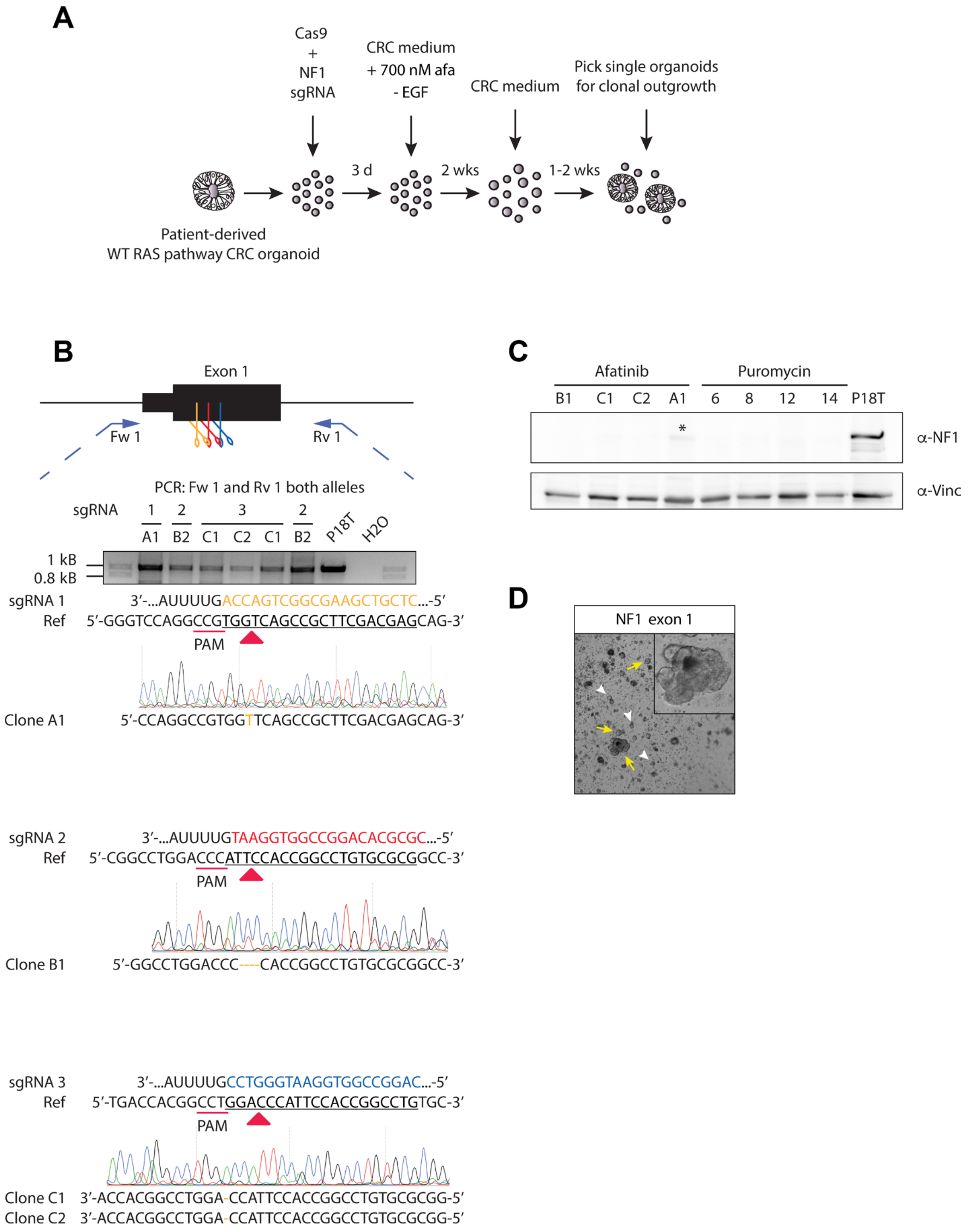

Supplementary Figure 3: Generation of CRISPR-mediated NF1 knock out in patient-derived CRC organoids using phenotypic selection. (**A**) Selection strategy to generate NF1 knock out organoids after CRISPR-induced small indel generation. (**B**) Genetic strategy for CRISPR/Cas9-mediated generation of small indels in exon 1 of *NF1* via non-homologous end-joining. The black box illustrates exon 1, and thin strokes illustrate introns. Scissors show double stranded breaks generated by three different sgRNAs (yellow, red and blue). Blue arrows illustrate PCR primer pairs. The agarose electrophoresis gel shows the PCR product of the targeted exon of *NF1* in selected clones. PCR amplification products were sequenced. Regions of sgRNA 1, 2 and 3 complementary to the protospacer (underlined) are shown in yellow, red and blue, respectively. Red arrow heads indicate cleavage sites and introduced mutations are shown in orange (**C**) Western blot analysis of NF1 expression in the indicated organoid lines. Left 4 clones are selected on phenotype (afatinib presence). For reference, right 4 clones are reproduced from Figure 3C. Asterisks indicate residual NF1 protein presence. (**D**) Representative picture of NF1 knock out organoids selected by EGF depletion and EGFR inhibition (afatinib).

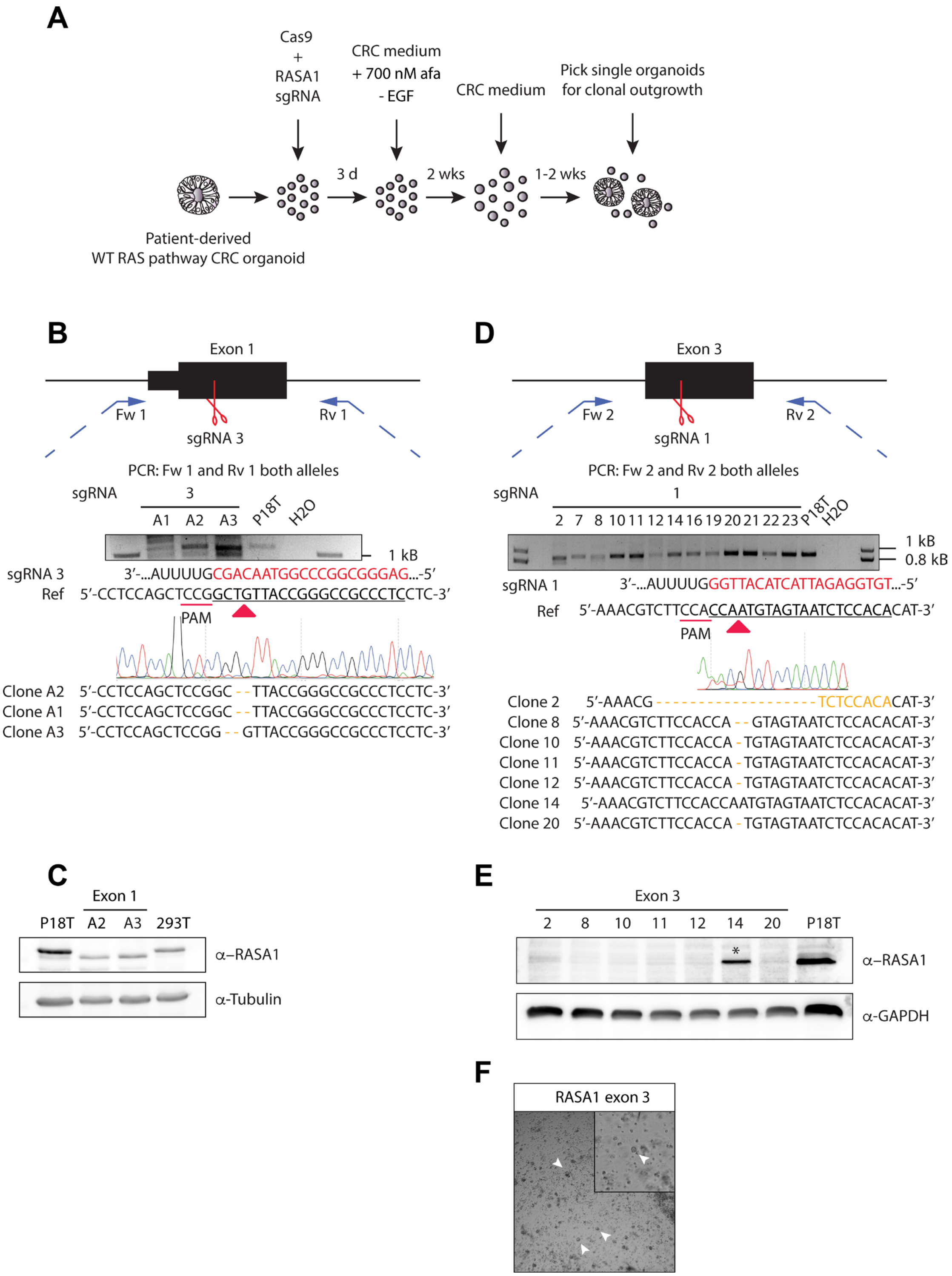

Supplementary Figure 4: Generation of CRISPR-mediated RASA1 knock out in patient-derived CRC organoids using phenotypic selection. (**A**) Selection strategy to generate RASA1 knock out organoids after CRISPR-induced small indel generation. (**B**, **D**) Genetic strategy for CRISPR/Cas9-mediated generation of small indels in exon 1 and 3 of *RASA1* respectively via non-homologous end-joining. The black boxes illustrate exon 1 and 3 respectively, and thin strokes illustrate introns. Scissors show double stranded breaks generated by different sgRNAs. Blue arrows illustrate PCR primer pairs. The agarose electrophoresis gel shows the PCR product of the targeted exons of *RASA1* in selected clones. PCR amplification products were sequenced. Regions of sgRNAs complementary to the protospacer (underlined) are shown in red. Red arrow heads indicate cleavage sites and introduced mutations are shown in orange Note that the *RASA1* exon 3 sequence of clone 14 reveals to be wild type. (**C**, **E**) Western blot analysis of RASA1 levels in the indicated organoid lines. Note that targeting of exon 1 of RASA1 results in predominant expression of a truncated isoform due to an alternative start site in exon 1. Asterisks indicate wild type RASA1 protein presence. In agreement with the wild type sequence of clone 14, normal levels of RASA1 is detected. (**F**) Representative picture of RASA1 exon 3 knock out organoids selected by EGF depletion and EGFR inhibition (afatinib).

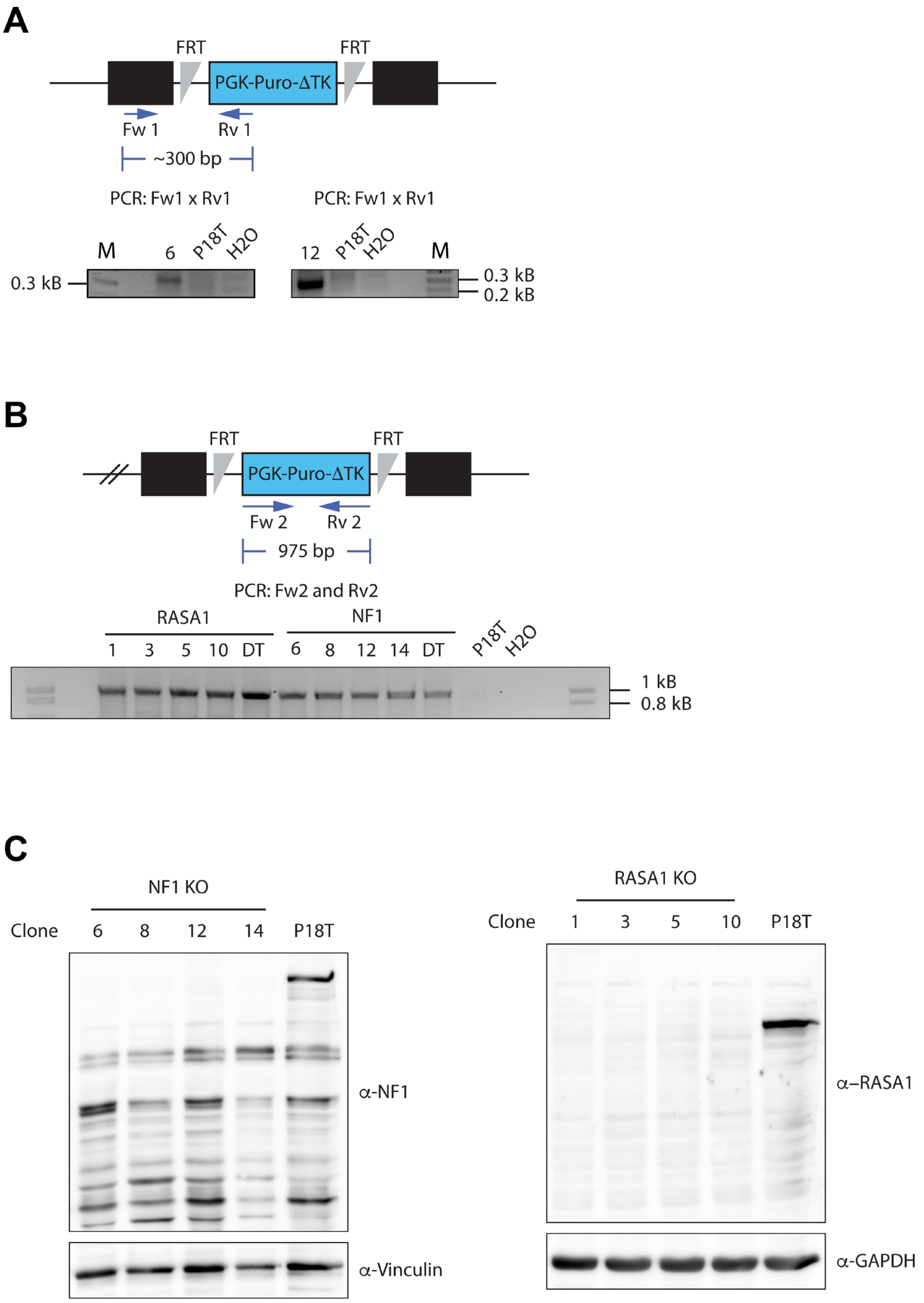

Supplementary Figure 5: Genomic integration of puromycin selection cassette in NF1 and RASA1 knock out patient-derived CRC organoids. (**A**) The structure of the targeted exon of *NF1* is depicted at the top. Black box illustrates exon 1, and thin strokes illustrate introns. Blue arrows illustrate PCR primer pairs. The agarose electrophoresis gel shows the ~300 bp PCR product of the integrated puromycin selection cassette at the targeted *NF1* locus in the selected clones (knock out clones # 6 and 12). (**B**) The structure of the targeted exon of *NF1* and *RASA1* is depicted at the top. The agarose electrophoresis gel shows the ~1 kb PCR product of the integrated puromycin selection cassette in the genome of the selected clones that were targeted for homologous recombination (RASA1 knock out clones # 1, 3, 5 and 10, and NF1 knock out clones # 6, 8, 12 and 14). The donor template (DT) was used as a positive control. (**C**) Uncropped images of western blot analysis for NF1 and RASA1 presence in the indicated organoid lines.

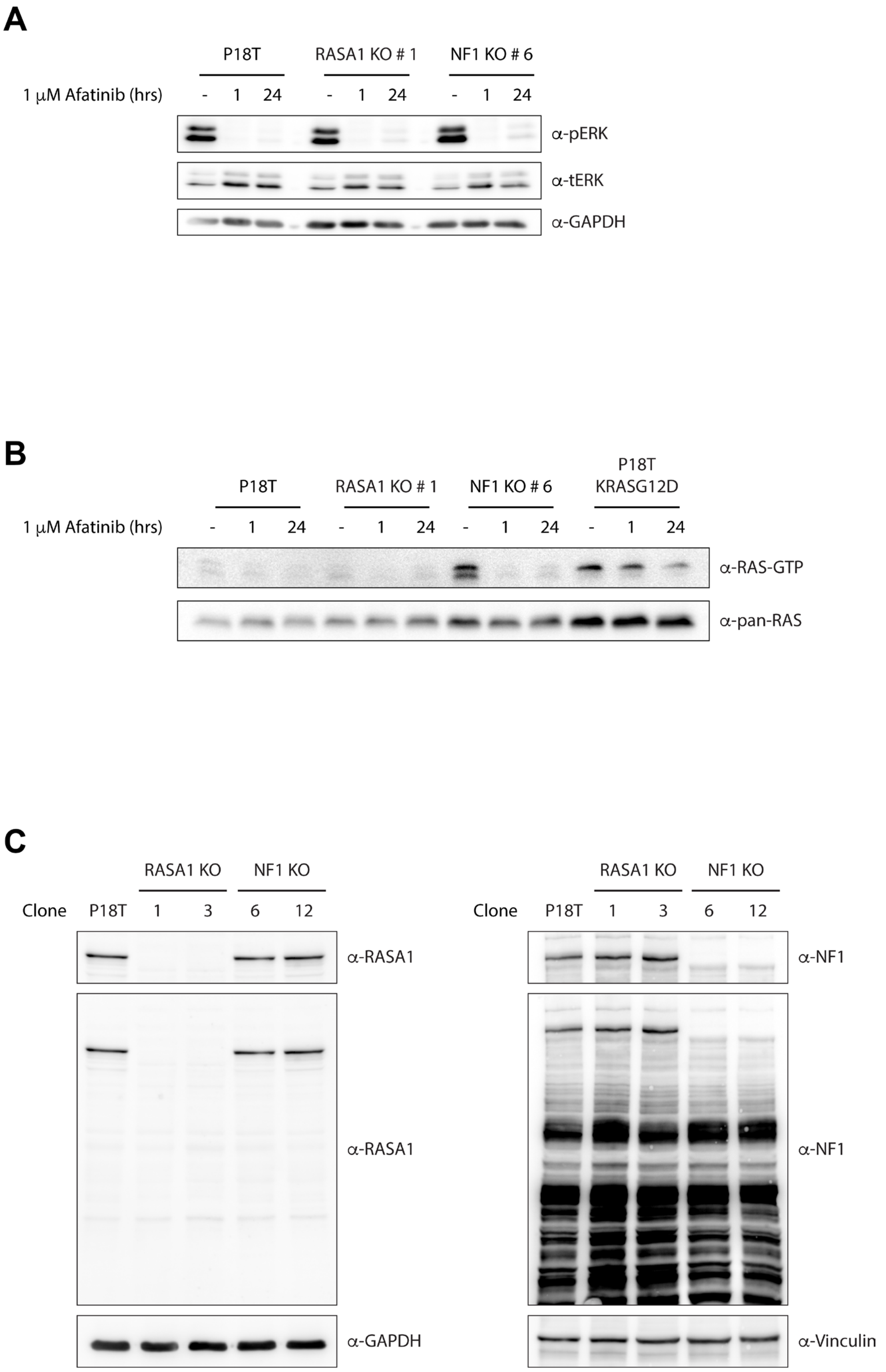

Supplementary Figure 6: Puromycin selected NF1 knock out CRC organoids show enhanced RAS and ERK activation. Same as Figure 5, but for different clones. (**A**) In comparison to P18T and P18T RASA1^{KO} (clone # 1), predominantly the P18T NF1^{KO} (clone # 6) shows enhanced basal ERK phosphorylation levels. Representative from n = 3 independent experiments. (**B**) Loss of NF1 (clone # 6) leads to elevated levels of RAS activity (GTP-loading) at basal conditions compared to P18T and P18T RASA1^{KO} (clone # 1) CRC organoids. The presence of an oncogenic mutation in KRAS (P18T KRAS^{G12D}) leads to elevated and sustained high levels of RAS activity (GTP-loading) at basal and in afatinib-treated conditions, respectively. RAS immunoblots from RAS pull-down assays are shown (RAS-GTP), together with a RAS immunoblot from total cell lysates as loading control. HRAS, KRAS, and NRAS isoforms are detected. Representative from n = 2 independent experiments. (**C**) Uncropped images of immunoblots of P18T, P18T RASA1^{KO} (clone # 1 and 3), P18T NF1^{KO} (clone # 6 and 12) CRC organoids indicate that the loss of RASA1 does not result in elevated protein levels of NF1, and vice versa. Representative from n = 3 independent experiments.

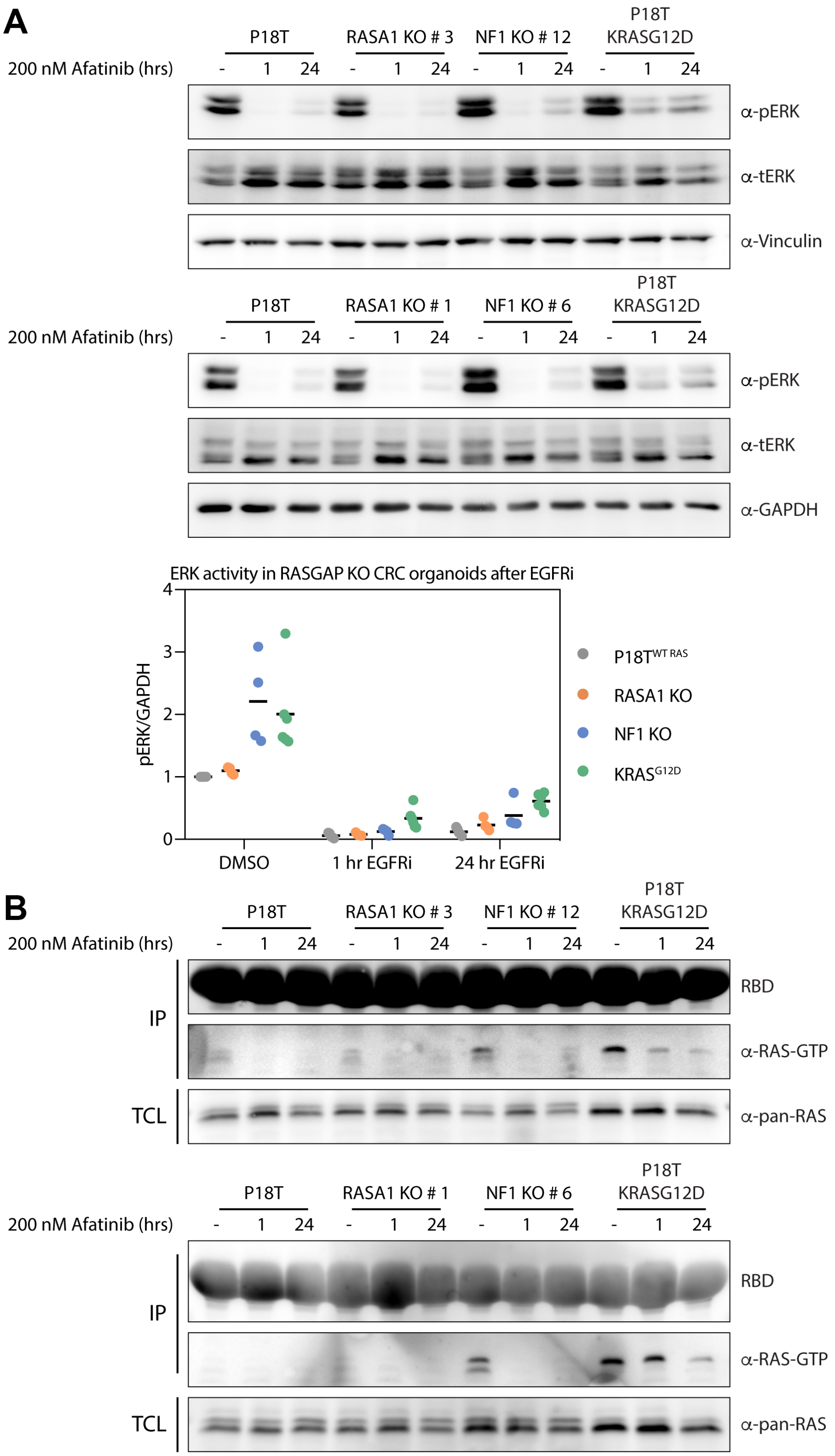

Supplementary Figure 7: Puromycin selected NF1 knock out CRC organoids show enhanced RAS and ERK activation (low concentration of EGFR inhibition). Same as Figure 5 and Supplementary Figure 6, but at 200 nM of afatinib treatment. (**A**) In comparison to P18T and P18T RASA1^{KO}, predominantly the P18T NF1^{KO} shows enhanced basal ERK phosphorylation levels. Representative from n = 2 independent experiments. Scatter dot plot depicts ERK phosphorylation levels normalized to GAPDH of P18T parental, RASA1^{KO} and NF1^{KO} organoids (*n* = 3). (**B**) Loss of NF1 leads to elevated levels of RAS activity (GTP-loading) at basal conditions compared to P18T and P18T RASA1^{KO} CRC organoids. The presence of an oncogenic mutation in KRAS (P18T KRAS^{G12D}) leads to elevated and sustained high levels of RAS activity (GTP-loading) at basal and in afatinib-treated conditions, respectively. RAS immunoblots from RAS pull-down assays are shown (RAS-GTP), together with a RAS immunoblot from total cell lysates as loading control. HRAS, KRAS, and NRAS isoforms are detected. Representative from *n* = 2 independent experiments.

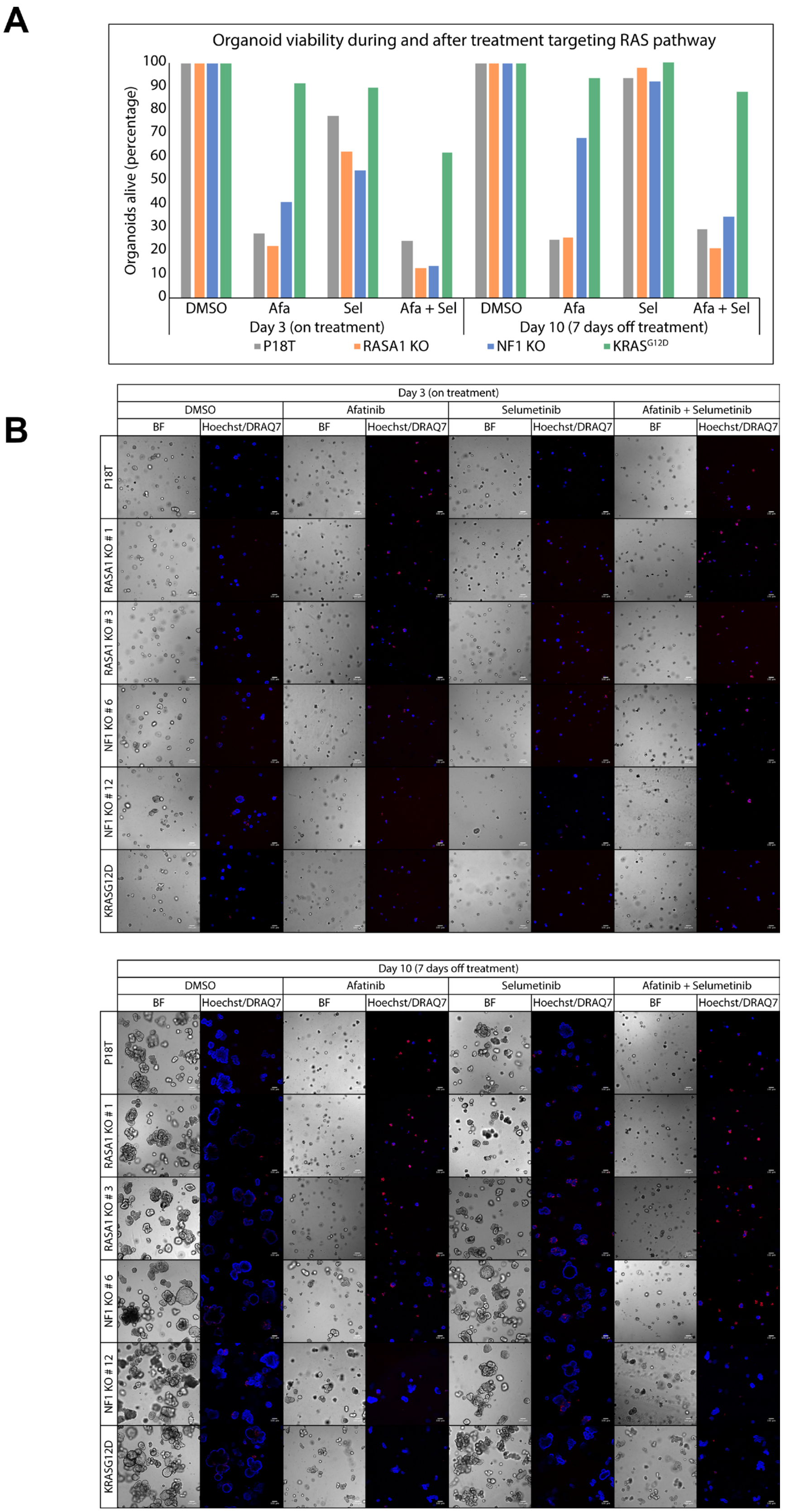

Supplementary Figure 8: Puromycin selected NF1 knock out CRC organoids show enhanced organoid survival and growth upon release of RAS-MAPK pathway inhibition. (**A**) Bar graph depicts the frequency of alive organoids after 3-day treatment and 7-day release of treatment with colorectal cancer (CRC) medium containing either DMSO, 1 µM afatinib (EGFRi), 1 µM selumetinib (MEKi), or a combination of 1 µM afatinib and 1 µM selumetinib in P18T parental, RASGAP knock out, and oncogenic mutant KRAS organoids. (**B**) Original pictures of organoids depicted in Figure 6. Scale bars, 100 µM. Hoechst and DRAQ7 was used to visualize nuclei and dead cells, respectively.

Supplementary Table 1: Target sites of and sgRNA guide sequences used for the generation of knock out organoids

Target site	Guide sequence
RASA1 exon 1	GAGGGCGGCCCGGTAACAGC
RASA1 exon 3	TGTGGAGATTACTACATTGG
NF1 exon 1 # 1	CTCGTCGAAGCGGCTGACCA
NF1 exon 1 # 2	CGCGCACAGGCCGGTGGAAT
NF1 exon 1 # 3	CAGGCCGGTGGAATGGGTCC
NF1 GAP domain	TCCATGCAGACTCTCTTCCG
RASA1 GAP domain # 1	CAAGTGTTGTGGCTCGAAAT
RASA1 GAP domain # 2	TGTGGCTCGAAATAGGGTAG
RASA2 GAP domain	GATGCAAACACAATTTTTAG
RASA3 GAP domain	CAGTGAGTTTCCTCGGAAGA
RASA4 GAP domain	CCAGAGAGTTGCTCCGGAAC
RASAL1 GAP domain	CCAGGGAGTTAGAACGGAAG
RASAL2 GAP domain	ATGTCTGAGGTGGATCGTTG
RASAL3 GAP domain	GCCGTGAGGCGCTGCTGTTC
SYNGAP1 GAP domain	GCGTGTTCTCGCGGAATATG
DAB2IP GAP domain	ACAACGAGCACCTCATCTTC
